# Improving the Performance of Paper-Based Dipole Antennas by Electromagnetic Flux Concentration

**DOI:** 10.1021/acsami.2c19889

**Published:** 2023-02-20

**Authors:** R. Carvalho, R. Brito-Pereira, N. Pereira, A. C. Lima, C. Ribeiro, V. Correia, S. Lanceros-Mendez, P. Martins

**Affiliations:** † Physics Centre of Minho and Porto Universities (CF-UM-UP), Universidade do Minho, 4710-057 Braga, Portugal; ‡ LaPMET—Laboratory of Physics for Materials and Emergent Technologies, Universidade do Minho, 4710-057 Braga, Portugal; § Centre for MicroElectroMechanics Systems (CMEMS), University of Minho, 4710-057 Braga, Portugal; ∥ BCMaterials, Basque Center for Materials, Applications and Nanostructures, UPV/EHU Science Park, 48940 Leioa, Spain; ⊥ IKERBASQUE, Basque Foundation for Science, 48009 Bilbao, Spain; # IB-S Institute of Science and Innovation for Sustainability, University of Minho, 4710-057 Braga, Portugal

**Keywords:** materials science, antennas, magnetic materials, energy materials, sustainability

## Abstract

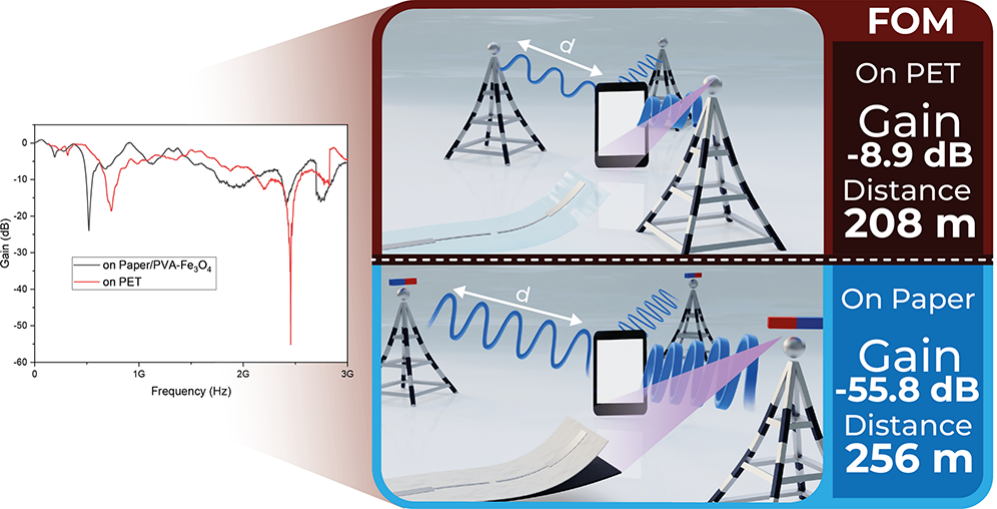

One of the essential issues in modern advanced materials science is to design and manufacture flexible devices, in particular in the framework of the Internet of Things (IoT), to improve integration into applications. An antenna is an essential component of wireless communication modules and, in addition to flexibility, compact dimensions, printability, low cost, and environmentally friendlier production strategies, also represent relevant functional challenges. Concerning the antenna’s performance, the optimization of the reflection coefficient and maximum range remain the key goals. In this context, this work reports on screen-printed paper@Ag-based antennas and optimizes their functional properties, with improvements in the reflection coefficient (*S*_11_) from −8 to −56 dB and maximum transmission range from 208 to 256 m, with the introduction of a PVA-Fe_3_O_4_@Ag magnetoactive layer into the antenna’s structure. The incorporated magnetic nanostructures allow the optimization of the functional features of antennas with possible applications ranging from broadband arrays to portable wireless devices. In parallel, the use of printing technologies and sustainable materials represents a step toward more sustainable electronics.

## 1 Introduction

The Internet of Things (IoT) technology represents the interconnection of the digital and physical worlds through appropriate information and communication technologies, enabling a large variety of new applications and services.^1^ In fact, the 5G’s connectivity will soon expand the mobile IoT, paving the way for rapid innovation across several industries and markets, including driverless cars, smart cities and homes, logistics monitoring, on-body wireless communication/energy harvesting, among others.^2^ By 2030, a multibillion IoT technology users are expected, thus the networks that provide digital–physical communications represent a great opportunity in the development of new advanced functional materials, shaping the ongoing digitalization of both society and economy.^3^

Smart sensors, smart antennas, and intelligent processors represent the backbone of IoT technology.^4^ Apart from the sophisticated sensing and processing platforms, the choice of an appropriate antenna systems is particularly critical for the successful implementation of the IoT solutions, as this component is responsible for sending/receiving the information generated by any source/sensor to the processing tools of the IoT.^4^

Several antenna designs that address specific IoT application challenges at different frequencies have been proposed. The reported solutions include compact and low-profile multistandard antenna designs with wide frequency coverage in the sub-6 GHz bands such as inverted-F antennas, loop antennas, and monopole antennas.^2^

Efficient power management, communication over long distances, lightweight, and flexibility to improve integration into specific applications are challenging requirements for different antenna types. Beyond those technological demands, environmental concerns are also affecting the way in which materials and devices are being/should be manufactured.^5^ To prevent the increasing problem related to electronic wastes (e-wastes) and reduce the impact of electronic processing technologies, a sustainable route for the IoT implementation must rely on more sustainable production techniques (such as additive manufacturing), materials, and substrates.^6,7^ Regarding the choice of the antennas’ substrates, cellulose is one of the most widely used, inexpensive, and commonly available materials and possesses excellent biodegradable properties.^8^ Additionally, cellulose-based paper is lightweight and can be processed for storage in small spaces or made into three-dimensional (3D) self-standing structures, being in this way a promising flexible substrate for IoT-related devices, including antennas.^9,10^ Cellulose is also compatible with the development of flexible electronics suitable for wearable and stretchable IoT applications.^11^ Nevertheless, to be fully compatible with such a flexible electronics concept, the integrated components also need to be highly flexible and mechanically robust, exhibiting also high tolerance levels in terms of cycling bending and thermal stability.^12,13^

The reflection coefficient *S*_11_ represents the amount of energy returning to the analyzer and is typically used to evaluate the antenna’s efficiency and tuning: a more negative *S*_11_ means that more amount of energy is being delivered to the antenna.^14−16^ Thus, *S*_11_ and maximum read distance/range remain the two biggest challenges in the development of new communication solutions.^17−22^ The introduction of a layer with high magnetic permeability to the traditional antenna’s structure allows to improve both antenna’s characteristics by causing the incoming electromagnetic radiation to flow through this layer, preventing the signal from reaching any conductive structures, technological devices, or animals/humans near the antenna. In addition, a soft ferrite layer can enhance the magnetic coupling/power transfer capability between the transmitting and receiving antennas^23^ as revealed by eq 1
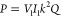
1where *P* is the power transferred, *V*_1_ and *I*_1_ are the voltage and current for the primary antenna, respectively, *k* is the coupling factor, and *Q* is the secondary quality.^24^

**Figure 1:**
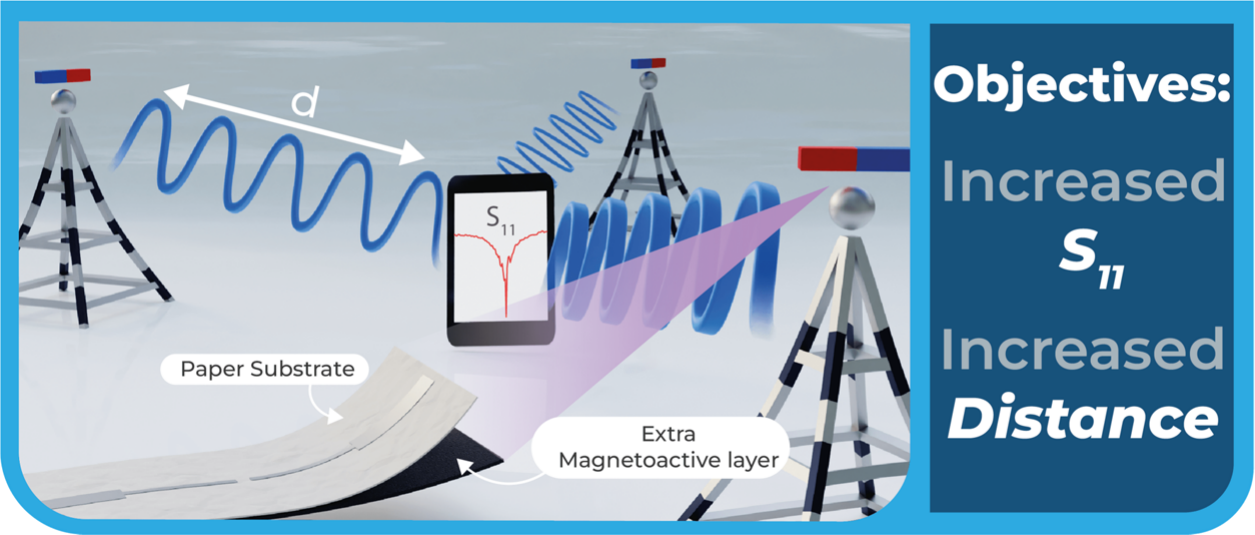
Objectives of the work: increase printed paper-based antenna’s *S*_11_ and maximum communication distance.

Thus, the present work focuses on the design and screen printing of a paper-based antenna with a magnetic Fe_3_O_4_-based layer with the aim to optimize both antenna’s *S*_11_ and maximum communication distance (Figure 1).

The magnetically optimized antenna has been printed both in polyethylene terephthalate (PET, one of the most used polymers in printed antenna-related applications^25−27^) and paper substrates with the aim to assess the influence of the substrate on the antenna’s performance.

Screen printing has been used to produce the antenna as it allows rapid and industrially scalable fabrication with reduced material waste. Further, screen printing offers a high deposition rate (deposited material weight per unit time), being of particular interest for the fabrication of communication devices with high areal capacitance.^28,29^ The antenna patch was screen printed with elastic conductive silver ink to be compatible with flexible electronics applications.^30^ Finally, the 2.4 GHz antenna band was selected to allow ZigBee, Bluetooth low energy (BLE), and WiFi applications. Those technologies are present in smart city scenarios and are very common in IoT applications.^31,32^

Fe_3_O_4_ nanoparticles have been chosen as magnetic components of the antennas due to their suitable magnetic response and the possibility to be obtained through sustainable green chemistry,^33,34^ and poly(vinyl alcohol) (PVA) was selected as a magnetic layer’s matrix due to its biodegradability and solubility in water.^35,36^

## 2 Materials and Methods

### 2.1 Materials

PVA was acquired from Sigma-Aldrich (Missouri), and Fe_3_O_4_ nanoparticles of ≈30 nm were acquired from Nanoamor (Texas). Melinex 506, 100 μm thick PET film and ME603 elastic conductive silver ink were both purchased from DUPONT (Wilmington). Origami paper (LOP) was obtained from IKEA-Ingka (Delft, Netherlands).

### 2.2 Preparation of PVA-Fe_3_O_4_ Magnetic Inks and the Corresponding Printing of the Films

The magnetic ink to be used for the printing of the magnetic layer is composed of PVA, Fe_3_O_4_ nanoparticles, and distilled water. Initially, Fe_3_O_4_ nanoparticles with a concentration of 90% in weight (wt %) were added to 6 mL of distilled water and 1 g of PVA powder under mechanical agitation at 90 °C until the complete dissolution of the polymer (≈2 h). Then, the solution was screen printed (DSTAR, model DX-305D with 250 mesh/inch) over both substrates (paper and PET). After the printing step, the samples were placed in an oven (JP Selecta, Model 2000208) with low humidity at room temperature (≈25 °C) for 24 h to ensure the complete evaporation of the water. Homogeneous films with an average thickness of ≈78 μm were obtained.

### 2.3 Printing of the Conductive Tracks

Dipole antennas were selected as they are being increasingly used in the framework of the Internet of Things (IoT) due to their low cost, low profile, ease of integration, lightweight, and good radiation performance.^37,38^

The different antenna geometries were printed using a DUPONT ME603 elastic conductive silver ink.

**Figure 2:**
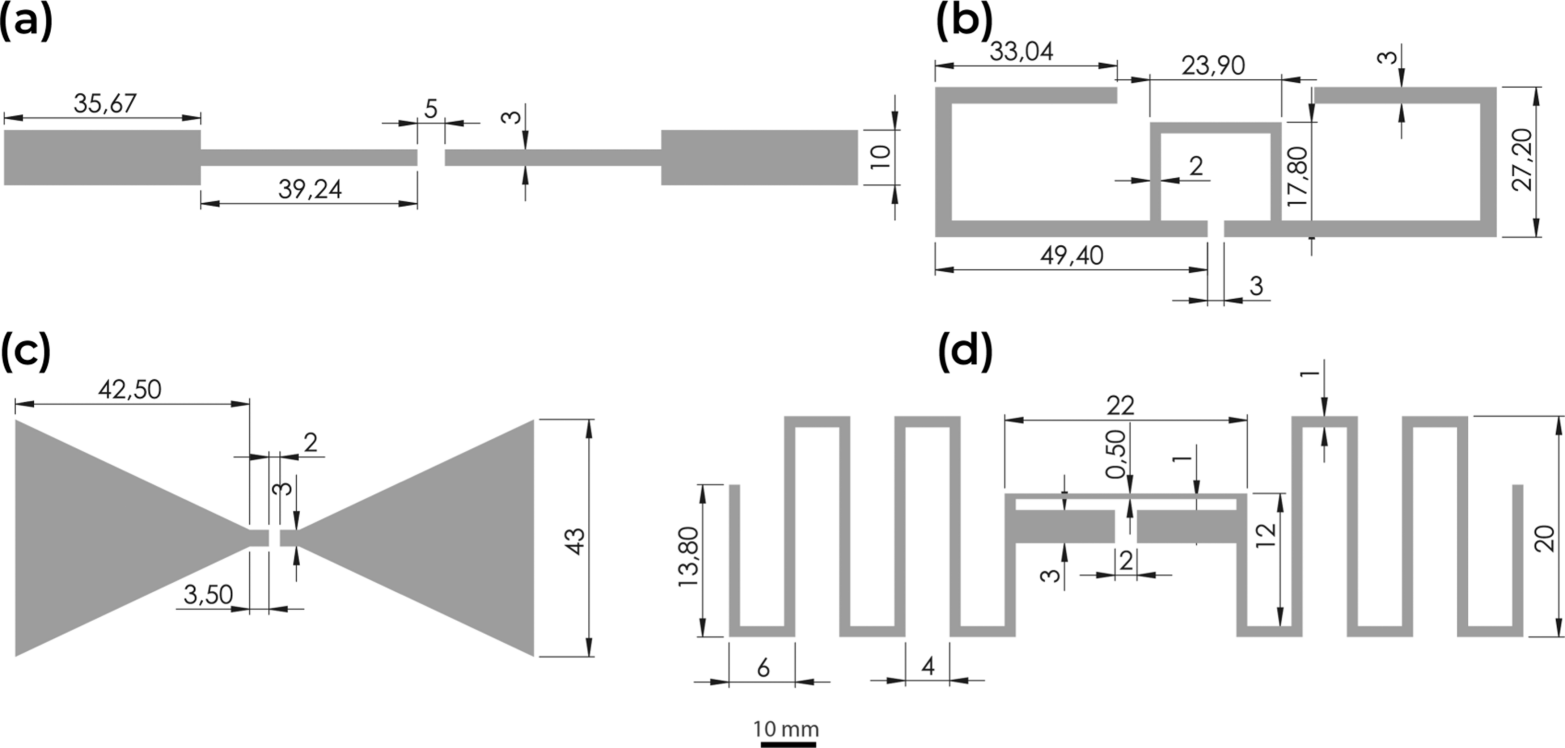
Printed geometries: (a) rectangular dipole antenna; (b) dipole antenna with an inductive loop; (c) triangular dipole antenna; and (d) winding dipole antenna with an inductive loop. Please note that the dimensions are in mm.

To prove the concept in a more representative way, the four most common dipole antenna geometries were fabricated: rectangular dipole antenna; (b) dipole antenna with an inductive loop; (c) triangular dipole antenna; and (d) winding dipole antenna with an inductive loop (Figure 2).

The stretchable conductive silver (Ag) ink was screen printed with a homemade system (with a vacuum table and adjustable speed for the printing squeegee rulers) with a 120-wire polyester mesh, reaching a final thickness of the printed patterns of ≈5 μm. Three different groups of samples were produced: (i) Ag printed on PET; (ii) Ag printed on PVA-Fe_3_O_4_, which in turn was previously printed on PET; and (iii) Ag ink and PVA-Fe_3_O_4_ printed on opposite sides of the PET substrate.

For the printing process, a stencil with 550 mm × 450 mm frame dimensions placed at a distance of 3 mm from the substrate and a printing velocity of 0.3 m·s^–1^, was used. After the printing process, the laminates (Ag+PVA-Fe_3_O_4_+substrate and Ag+substrate) were placed in an oven (JP Selecta, Model 2000208) for 1 h at 90 °C for thermal curing of the silver ink. After this thermal treatment, the magnetoactive antennas were ready to be tested for communication.

### 2.4 Materials and Printed Layer Characterization

Rheological characterization was carried out with an AresG2 rheometer with a 40 mm flat plate geometry and a 1 mm gap. The mixture was placed onto the rheometer plate immediately after the complete dissolution of the polymer and suitable dispersion of the magnetic particles was obtained. Flow curves were acquired by a three-shear rate sweeps program (up–down–up), setting a nonstop ramp and a 0 to 300 s^–1^ shear rate. The apparent viscosity at 3 s^–1^ was studied for the unsteady state (curve 1) once for this condition the structure was less disturbed. All other shear rate values (steady state) were evaluated from curve 3. The determined viscosity (η) value for the developed ink was evaluated at distinct shear rates (γ), assuming a power-law model^39,40^ (eq 2)
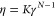
2where the coefficients *N* and *K* are experimentally determined.

Knowing that the surface properties of a substrate are among the most important parameters in the printing of functional materials, determining not only the printing resolution but also the stability of the printed features,^41^ contact angle measurements of the different samples were performed using a Data Physics OCA20 instrument by the static sessile drop method using different testing mixtures/liquids: PVA-FO ink, Ag ink, and water. For that, 3 μL drops of different samples were left on the surface of the different substrates (paper, PET, and PVA-FO) and the contact angles were determined using the SCA20 software. The mean contact angle and standard deviation were obtained after measurments at six different locations.

The morphology of the developed materials was evaluated by scanning electron microscopy (SEM) using a NanoSEM-FEI Nova 200 (FEG/SEM) scanning electron microscope (10 kV). Before experiments, all samples were coated with Au with a Polaron SC502 sputter coater. The thickness of the layers was calculated from five images with 15 measurements in each image using the ImageJ software.

Adhesion of the printed inks was evaluated with an adapted tape peel test^42^ carried out on samples of size 1 cm × 1 cm. Briefly, an adhesive tape (3 M Scotch Magic tape 810) was pressed on the surface of the printed samples with different forces (100, 200, 300, and 400 N) for 30 s using a Shimadzu AG-IS universal test setup in compression mode at 2 mm·min^–1^. After that, the tape was removed from the sample at the same velocity while monitoring the required force. The different samples were weighed before and after each test to determine the mass loss in each experiment.

The samples’ DC surface electrical conductivity was obtained by measuring the characteristic IV curves at room temperature with a Keithley 6487 picoammeter/voltage source. Previously, two rectangular electrodes (4 mm length x 1 mm width, with a spacing of 3 mm) were deposited using a Polaron, model SC502 sputter coater. From the IV characteristics of the samples, the electrical resistivity (ρ) was determined, considering the geometrical characteristics according to eq 3
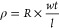
3where *R* is the film resistance, calculated by the inverse of the slope of the IV data, *l* is the distance among Ag electrodes, *w* is the length of the Ag electrodes, and *t* is the sample’s thickness, measured with a Digimatic Micrometer MDC-25PX. The electrical conductivity σ was then determined as ρ^–1^.

Magnetization M(H) curves were obtained at room temperature up to 1.85 T using a MicroSense EZ7 vibrating sample magnetometer. From these loops, saturation magnetization and coercive field of the composites were obtained.

To evaluate the influence of an applied magnetic field on the paper-based antennas, an external bias field was applied (in-plane and out-of-plane to the plane of the antenna’s surface) by an electromagnet with a maximum value of 0.4 T.

### 2.5 Antenna Characterization

For the evaluation of the antenna performance, a Received Signal Strength Indicator (RSSI) analysis (that measures the amount of power in a signal) was performed as a function of distance and time. This is an accurate strategy to evaluate both signal strength and communication quality. To this end, a starting point (P_0_) common to all tests was defined, and from this point, through the APPBLE Scanner software, the signal reception power was measured, on a REDMI Note 7 mobile phone, as a function of the distance to P_0_. One of the most used commercial antennas, ANT-W63RPC1-MHF4-50, was used for device performance comparison.

## 3 Results and Discussion

### 3.1 Materials and Layers

**Figure 3:**
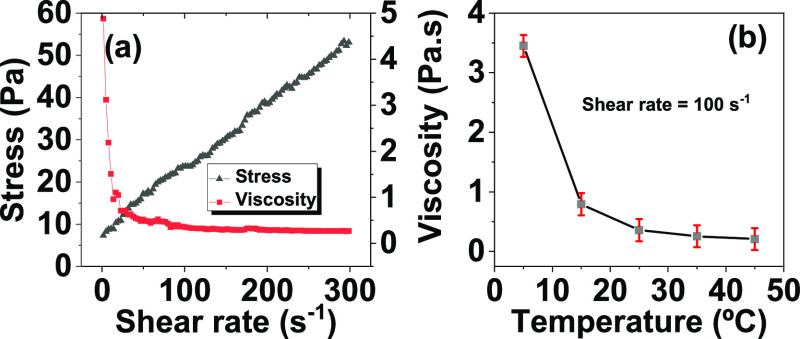
(a) Room-temperature shear stress and ink viscosity vs shear for the PVA-Fe_3_O_4_·H_2_O ink. (b) Viscosity as a function of temperature for the PVA-Fe_3_O_4_·H_2_O ink.

Since the viscosity of ink determines its printing conditions, being the most important rheological characteristic that influences the printing resolution,^41^ rheological measurements were carried out on the magnetic PVA-Fe_3_O_4_·H_2_O inks, as presented in Figure 3.

Previous reports revealed that the inks for the screen printing process should show pseudoplastic behavior, which displays a decreasing viscosity with an increasing shear rate, and also a thixotropic behavior (as the shear rate, which translates to the combination of squeegee pressure, velocity, and screen tension, is increased, the paste becomes substantially thinner, causing it to flow more readily).^43^

Figure 3a reveals such a pseudoplastic behavior of the PVA-Fe_3_O_4_·H_2_O ink: when the shear rate is applied, the ink components are rearranged to accommodate the shear rate. As a consequence, the overall shear force is smaller for lower shear rates as a result of the lower response time of the inks, whereas for the minor shear rates, the ink constituents have sufficient time to reorder; therefore, increasing shear rates lead to lower reorganization times and higher induced stress.^44^ Increased temperatures (Figure 3b) at a shear rate of 100 s^–1^ lead to a decrease in the viscosity of the ink as a result of the increased thermal motion of the polymer chains and hindered intermolecular physical interactions between polymer chains, water, and magnetic nanoparticles.^45,46^ The obtained results demonstrate that the developed ink is within limits (up to 5 Pa·s) suitable for screen printing.^47^

To evaluate the PVA-Fe_3_O_4_·H_2_O and Ag inks spreading on different substrates, essential for successful printing,^48,49^ and to test the affinity for water (high affinity can lead to defective and heterogeneous PVA-Fe_3_O_4_ printed layers, once PVA dissolves in water), contact angle measurements have been performed (Table 1).

**Table 1:**
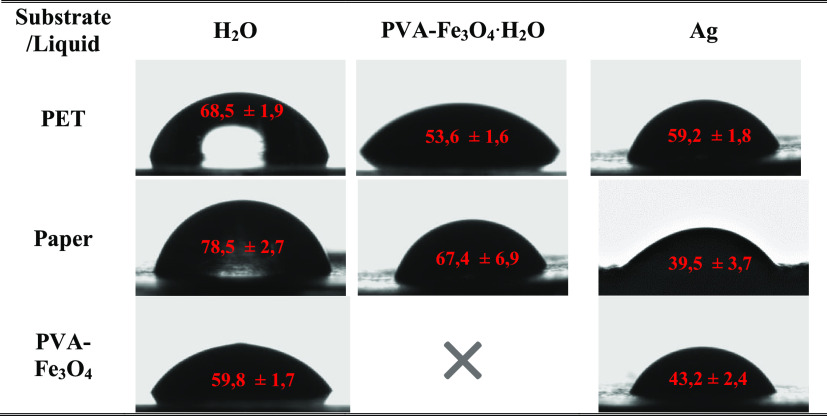
Contact Angle of Inks and Water on the Different Substrates Used for Device Development

In printing applications, the resolution and stability of printed features strongly depend on the contact angle.^41^ Regarding the contact angles obtained for the PVA-Fe_3_O_4_·H_2_O and Ag functional inks, both stay in the ≈40 to ≈70° range, independent of the substrate, which is suitable for improved wetting, allowing suitable drop spreading and delivering high printing quality.^41,48^ Smaller contact angles would result in overspill and overspreading of the ink, while larger contact angles could lead to the formation of mechanical instabilities and poor spreading.^41^ The water contact angle values also demonstrate that all samples are characterized by low hydrophobicity, which also facilitates the printing process.^50^

The contact angle of H_2_O of ≈60° on the PVA-Fe_3_O_4_ layer is higher than the one found on pristine PVA and similar to hydrophobically modified PVA-based devices.^51,52^

**Figure 4:**
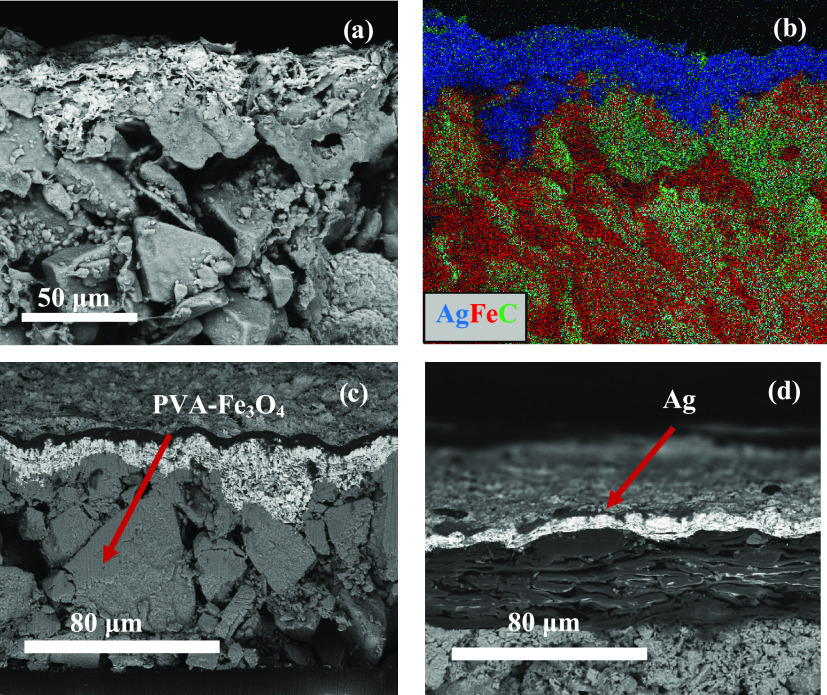
(a) Representative SEM image of the conducting silver layer printed on the PVA-Fe_3_O_4_ magnetoactive layer. (b) Color map obtained by EDS in the same sample and location of (a). Representative SEM images of different magnifications were used to determine the thickness of (c) PVA-Fe_3_O_4_ layers and (d) Ag layers.

SEM (Figure 4) was used to evaluate the morphology of the printed materials and quantify the thickness of each layer (Figure 4c,d). Elemental mapping analysis (energy-dispersive spectroscopy, EDS) was also conducted to evaluate the element distribution along the Ag and PVA-Fe_3_O_4_ active layers (Figure 4b).

SEM images show that both active layers remain continuous, homogeneous, and independent, which is a requirement for each of them to correctly fulfill their function of conductive (Ag) and magnetically responsive (PVA-Fe_3_O_4_) layers in the device. From the SEM images (Figure 4c,d) the thicknesses of the different layers were obtained: PVA-Fe_3_O_4_ = 78 ± 3 μm and Ag = 4.6 ± 1 μm. Despite all samples being cut in liquid nitrogen, the existing distortions and cavities are attributed to the mechanical deformations induced during the cutting procedure. The elemental mapping analysis (Figure 4b) confirmed the presence of Ag particles on top of the structure and magnetic Fe_3_O_4_ nanoparticles (Fe locations) in the PVA-Fe_3_O_4_ layer and, despite the high Fe_3_O_4_ content (90 wt %), the printing process ensured uniform distribution of the fillers in the sample.

**Figure 5:**
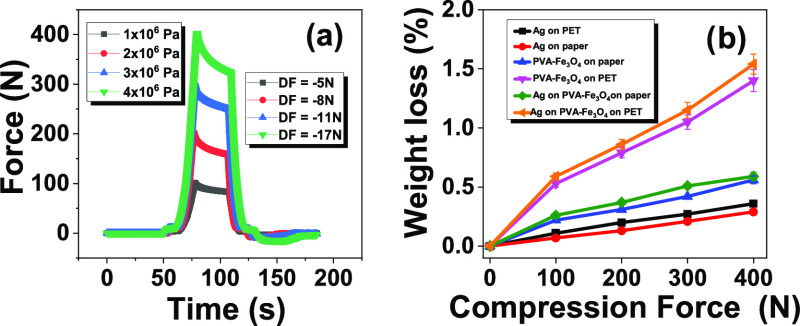
(a) Evolution of the applied force over time on a PET substrate for the four pressures applied. (b) Weight loss of each sample as a function of the force applied force. The peeling force was not dependent on the sample type (differences less than 5%).

To study the adhesion of the printed layers to the different substrates, peeling tests (Figure 5) have been performed.

Figure 5a shows that the peeling force is proportional to the compression force, with the highest compression force (400 N) leading to the highest peeling force (17 N) and the lowest compression force (100 N) leading to the lowest peeling force (5 N). Additionally, the resulting mass loss is proportional to the force applied by the load cell in all samples (Figure 5b). The results including PVA-Fe_3_O_4_·H_2_O printed on PET (Ag printed on PVA-Fe_3_O_4_ previously printed on PET and PVA-Fe_3_O_4_·H_2_O printed on PET) exhibit the highest mass loss (≈1.4%); the Ag layer printed on paper and on PET exhibit the lowest weight loss (≈0.2%); and the layers that correspond to PVA-Fe_3_O_4_·H_2_O printed on paper (Ag printed on PVA-Fe_3_O_4_ previously printed on paper and PVA-Fe_3_O_4_·H_2_O printed on paper) exhibit intermediate mass loss of ≈0.4%. In a second round of tests, none of the samples experienced a significant weight loss (less than 0.1%), showing the stability of all printed layers, independent of the layer and the substrates. Those values are compared favorably with ones reported on the efficient printing of metal nanoparticle inks into polymeric substrates.^53^

**Figure 6:**
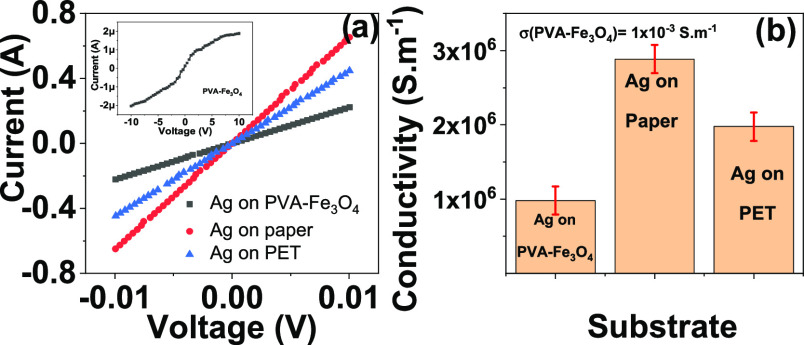
(a) Electric current variation as a function of applied voltage for the Ag layer printed on different substrates. The inset shows the electric current variation as a function of applied voltage for the PVA-Fe_3_O_4_ layer. (b) Electric conductivity values obtained from (a).

**Figure 7:**
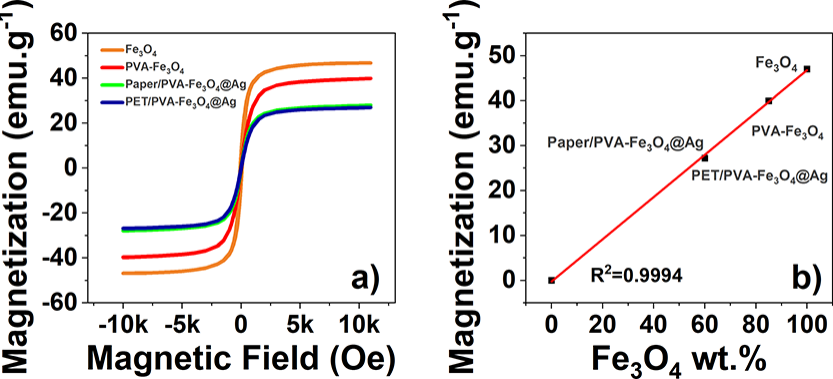
(a) Magnetization cycles of the different samples (Fe_3_O_4_ nanopowder, PVA-Fe_3_O_4_ layer, paper/PVA-Fe_3_O_4_@Ag layered structure, and PET/PVA-Fe_3_O_4_@Ag layered structure) as a function of the applied DC magnetic field. (b) Relation between Fe_3_O_4_ wt % present in a given sample and its saturation magnetization.

The functional characteristics of the different layers, i.e., electrical conductivity for the Ag printed layer and magnetic response for the PVA-Fe_3_O_4_ layer, are presented in Figures 6 and 7, respectively.

The representative *I*–*V* plots (Figure 6a) reveal a typical ohmic behavior in all samples, with the electric current increasing linearly with the applied voltage. The inset of Figure 6a shows less ohmic behavior on the PVA-Fe_3_O_4_ layer as a result of different contributions to conductivity from the composite’s different components (polymer, particles, and interfaces): PVA has higher resistance than Ag and the defective structure of the polymer resulting from the addition of Fe_3_O_4_ affects the electrical transport, dynamically rearranging the conductivity process under the current flow.^54,55^

The slope of the *I*–*V* plots and eq 3 allowed us to determine the DC surface electrical conductivity (Figure 6b). The porosity of the paper substrate resulting from its fibrilar structure led to a lower contact angle of the Ag drops (Table 1), which promoted more uniform printing patterns, higher interactions between substrate and printed layer, and more compact structures, ensuring a higher electrical conductivity of the Ag layers printed on paper substrates.^56,57^ The conductivity values of the Ag printed layers (1 × 10^6^ to 3 × 10^6^ S·m^–1^) are in the same order of magnitude as the ones recently reported on high-conductivity screen-printable silver nanowires for optically transparent flexible radiofrequency wireless communication.^58^

Additionally, the resistive behavior of the PVA-Fe_3_O_4_ (1 × 10^–3^ S·m^–1^) layer ensures improved electromagnetic performance of antennas by increasing their gain/*S*_11_.^59^

As the introduction of the magnetoactive layer on the antenna structure is expected to improve its performance, the magnetic characterization of the composite is performed through a vibrating sample magnetometer (VSM) (Figure 7).

All samples reveal the absence of hysteresis, remanence, and coercivity, consistent with the single domain behavior, as room temperature (≈30 °C) is above the blocking temperature and the nanoparticle’s magnetic moment is able to rotate in response to the imposed DC magnetic field.^60,61^ Additionally, the magnetic saturation of the PVA-Fe_3_O_4_ layer (≈39.5 emu·g^–1^) corresponds to 84.4% of the magnetic saturation of pure Fe_3_O_4_ (≈46.8 emu·g^–1^), thus showing that the printing process did not substantially affect the wt % of Fe_3_O_4_ in the PVA matrix. Additionally, magnetic measurements allowed us to determine the content of Fe_3_O_4_ on the multilayer structures, paper/PVA-Fe_3_O_4_@Ag, and PET/PVA-Fe_3_O_4_@Ag (≈58 wt %).

### 3.2 Antennas

Four different antenna geometries, rectangular dipole antenna, triangular dipole antenna, dipole antenna with inductive loop, and winding dipole antenna with inductive loop, were printed on PET substrates to determine which of them exhibited the highest *S*_11_ and the better 2.4 GHz tuning (used in ZigBee, BLE, and WiFi communications). Three different groups of samples were produced: (i) Ag printed on PET; (ii) Ag printed on PVA-Fe_3_O_4_, which in turn was previously printed on PET; and (iii) Ag ink and PVA-Fe_3_O_4_ printed on opposite sides of the PET substrate.

**Figure 8:**
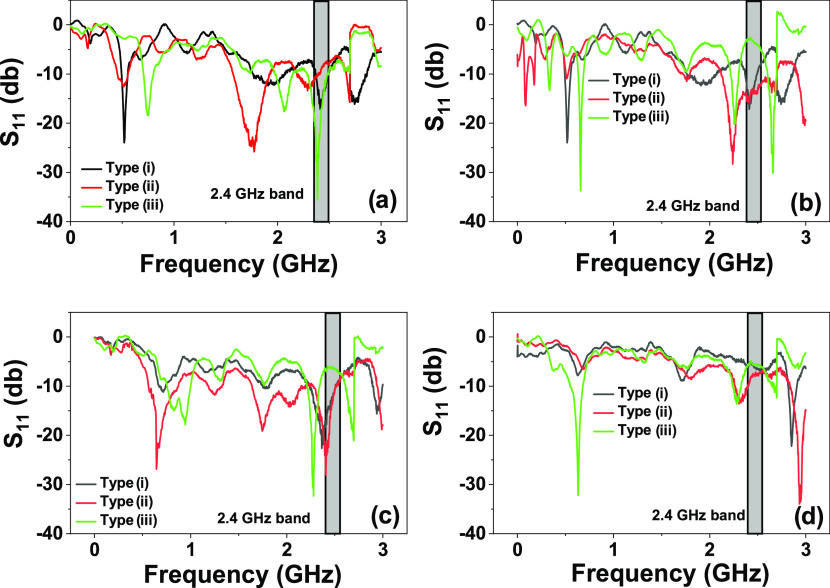
Analysis of the *S*_11_ (dB) as a function of frequency (GHz) for the different antenna geometries: (a) rectangular dipole; (b) dipole with inductive loop; (c) triangular dipole; and (d) winding dipole with inductive loop.

Figure 8 shows that the antenna geometry that exhibited higher gain and better 2.4 GHz tuning was the rectangular dipole type iii (−35 dB gain and 2.39 GHz frequency); the dipole with inductive loop and winding dipole with inductive loop antennas showed the worst performance (≈0.4 GHz drift on tuning); and the triangular dipole antenna revealed an intermediate feature. Such optimum tuning of the rectangular dipole antenna at 2.4 GHz is explained by the conjugation between the dielectric constant of the substrate and the geometry of the printed pattern.^62,63^Figure 8a also reveals that despite both samples ii and iii having a PVA-Fe_3_O_4_ layer in their composition, the different dielectric properties of the material on which the Ag layer was printed (ii: PVA-Fe_3_O_4_ and iii: PET) led to a different tuning (ii ≈1 GHz and iii ≈2.4 GHz).^64,65^

**Figure 9:**
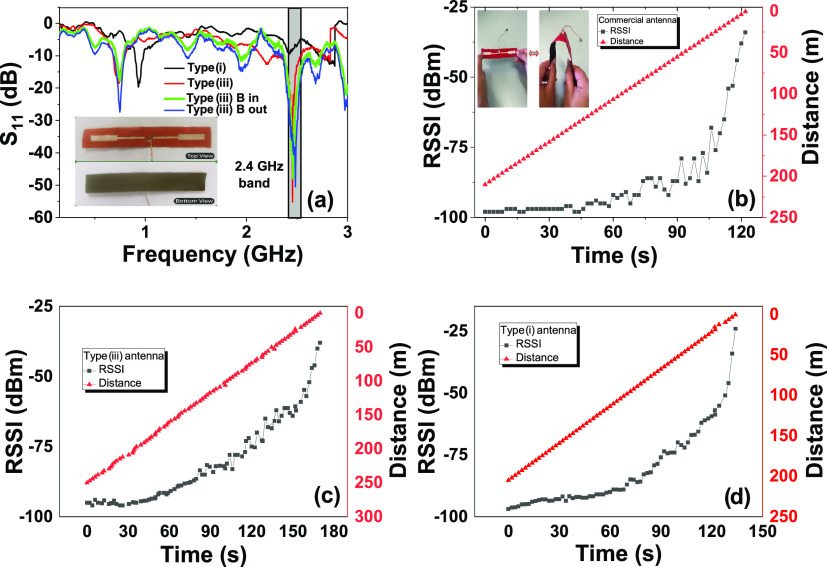
(a) Analysis of the *S*_11_ (dB) as a function of frequency (Hz) for the paper-based rectangular dipole antenna (type i and type iii). Panel (a) also exhibits the influence of a 0.4 T in-plane magnetic field (B) on the performance of the antenna. The inset shows the top view and bottom view of a printed paper-based antenna (type iii). Analysis of RSSI (dBm) as a function of time (s) and distance (m) for (b) commercial antenna (the inset shows the bending procedure to which paper-based antennas were subjected: 50 release/bending sequences); (c) type iii rectangular dipole antenna; and (d) type i rectangular dipole antenna.

Similarly, the small decrease of the *S*_11_ on type ii antennas (from −16.3 to −12.2 dB) when compared to type i antennas emerges from the lower conductivity of Ag when printed on PVA-Fe_3_O_4_. The high increase in gain (from −16.3 to −35.7 dB) of type iii antennas results from the higher conductivity of Ag on paper and from the tuning effect of the PVA-Fe_3_O_4_ layer that works as electromagnetic flux concentrators and successfully shields the canceling magnetic fields coming from the neighboring sources.^23,63^ The results from Figure 8 allowed us to select a rectangular dipole to develop paper-based devices, whose functional evaluation is represented in Figure 9.

Figure 9 reveals that type iii paper-based antennas exhibit a perfect tuning to the 2.4 GHz band and that the introduction of the Fe_3_O_4_ layer increased the gain to ≈500% (from −8.9 to −55.8 dB). Once again, the electromagnetic flux concentration, increased magnetic permeability, and successful shielding of the canceling magnetic fields coming from the neighboring sources explain this increase. All tested antennas exhibited a similar RSSI *vs* time behavior; nonetheless, the introduction of the PVA-Fe_3_O_4_ layer increased the maximum read distance in ≈20% (from 208 to 256 m), a fact that is explained by its optimized gain.^66^ Additionally, no substantial differences were detected when a DC magnetic field (0.4 T) was applied to the antenna’s surface: the tuned frequency changed from 2.44 to 2.46/2.48 GHz (in-plane/out-of-plane) and the *S*_11_ varied from −57 to −48/–50 dB (in-plane/out-of-plane). Such a variation is explained by the fact that the applied DC magnetic field greatly changes the permeability of the printed antenna, decreasing it with increasing magnetic field until the magnetic resonance occurs, which in turn increases the tuned frequency and decreases the *S*_11._(67)

Such maximum read distance of the proposed paper/PVA-Fe_3_O_4_@Ag antenna is even higher than the one found on the commercial antenna (215 m). Those values are also favorably compared with the ones recently reported on flexible and printed antennas (Table 2) and do not change after 50 release/bending sequences.

**Table 2:** Comparison between the Performances of the Proposed Antennas with Some Recently Reported Ones^b^

antenna	substrate	conductor	*S*_11_ (dB)	distance (m)	bandwidth^a^ (GHz)	refs
commercial ANT-W63RPC1-MHF4-50				215	2.400–2.485	(51)
planar monopole	paper	Ag	–15		2.2–2.7	(68)
multiband			–25		1.7–2.7	(69)
dipole	Taconic RF		–45		0.940–1.094	(70)
rectangular dipole	paper	Ag	–8.9	208	2.35–2.50	this work
	paper/Fe_3_O_4_		–55.8	256		

a *S*_1_ < −11 dB.

b Supporting Information shows a video that demonstrates the wireless transmission of data/energy over distance.

## 4 Conclusions

A flexible and low-cost multilayered screen-printed antenna with a size of 20 mm × 160 mm was fabricated on a commercial paper substrate. The different layers include origami paper or PET, PVA-Fe_3_O_4_, and Ag; and the fabricated antenna geometries are rectangular dipole antenna, dipole antenna with an inductive loop, triangular dipole antenna, and winding dipole antenna with an inductive loop. The range and *S*_11_ values have been studied as a function of frequency, substrate type, and antenna geometry. Overall, the best results are obtained for the rectangular dipole antenna geometry. The incorporation of a PVA-Fe_3_O_4_ layer on the antenna’s structure increased the *S*_11_ from −8 to −56 dB and the maximum range from 208 to 256 m.

The use of screen printing technology on a paper substrate will reduce the overall cost of the antenna and increase its sustainability, being compatible with mass production. The antenna can be easily optimized to operate at different frequency bands and therefore can be tailored for a wide range of applications.

## Supporting Information Available

Smartphone was connected by Bluetooth with an ESP32 module through the printed antenna, a LED was connected to the output pin of the ESP32 that toggles between on and off when a message from the smartphone is sent, at the same time, ESP32 sends a verification message to the smartphone to test the connection (“OK”) (MP4)
